# Associations Between Mukbang Watching and Appetite, Nutrition, and Quality of Life in Pediatric Patients with Cancer: Intensive Longitudinal Study

**DOI:** 10.2196/80932

**Published:** 2026-05-22

**Authors:** Yuxue Xiao, Jiani Wang, Ting Zhong, Xiao Wang, Dongyan Tang, Wei Xia

**Affiliations:** 1 School of Nursing, Sun Yat-sen University, RM 613, Block of School of Nursing, Sun Yat-Sen University North Campus, No. 74, 2nd Yat-Sen Road,Yuexiu District, Guangzhou, 510080, China, 86 020-87334851; 2 School of Nursing, Li Ka Shing Faculty of Medicine, The University of Hong Kong, Hong Kong, Southern, China (Hong Kong); 3 Department of Pediatric Hematologic Oncology, Sun Yat-sen Memorial Hospital, Guangzhou, China; 4 Department of Gerontology, Peking University Sixth Hospital, Beijing, China

**Keywords:** appetite, mukbang, nutrition, pediatric oncology, quality of life

## Abstract

**Background:**

Malnutrition is prevalent among children with cancer and is often exacerbated by diminished appetite. To combat this, hospitalized children are increasingly adopting mukbang, a popular online eating show genre.

**Objective:**

This study aimed to assess the associations between mukbang watching and appetite, nutrition, and quality of life in children with cancer.

**Methods:**

From September 2022 to June 2023, an intensive longitudinal study involved 179 children undergoing radiotherapy or chemotherapy at 2 tertiary hospitals in Guangzhou, China. Daily mukbang watching and appetite data were collected for 5 consecutive days. Nutrition and quality of life were assessed pretreatment and posttreatment. Latent class analysis and generalized estimating equation (GEE) models were used. Sensitivity analyses were conducted by stratifying participants based on baseline appetite levels using adjusted GEE models.

**Results:**

Among participants, 63.7% (114/179) watched mukbang during hospitalization, forming low (C2), medium (C3), and high (C4) watching classes. The adjusted appetite GEE model indicated a significant interaction effect of time × class in both the medium (β=0.08, 95% CI 0.02‐0.13; *P*=.006) and high classes (β=0.10, 95% CI 0.04‐0.17; *P*=.003). No significant association was observed between mukbang watching and nutritional status over time. A significantly higher quality of life was observed in the medium (β=19.18, 95% CI 7.84‐30.53; *P*=.001) and high (β=13.63, 95% CI 1.15‐26.12; *P*=.03) classes compared to the never class, with significant negative interactions of time × class observed in the low (β=−7.58, 95% CI −14.78 to −0.37; *P*=.04) and medium classes (β=−14.00, 95% CI −22.06 to −5.94; *P*=.001). Sensitivity analyses confirmed that appetite maintenance was robust across baseline appetite subgroups.

**Conclusions:**

Medium- and high-frequency mukbang watching were associated with better appetite maintenance over time among children undergoing treatment. Medium and high classes showed higher overall quality of life compared to the never class; however, the low and medium classes exhibited greater declines over time. These findings suggest the potential for mukbang in appetite-related symptom management while highlighting the need to address the psychological gap between virtual stimulation and physical reality in future research.

## Introduction

Globally, the incidence of pediatric cancer is increasing, with approximately 400,000 children and adolescents aged 0 to 19 years diagnosed with cancer worldwide [1]. Significant advancements in diagnosis, treatment, and nursing care have raised the average cure rate to 52% and the 5-year survival rate to 80% for children with cancer [2]. Standard cancer treatments, such as surgery and radiation, cause side effects that impact the physical, psychological, and social functioning of pediatric patients with cancer [3]. Consequently, these patients often experience compromised well-being and a reduced quality of life [3].

Evidence shows that malnutrition, particularly inadequate nutrition, can lead to decreased treatment tolerance, impaired functional capacity, prolonged hospital stays, and substantially reduced quality of life [4]. Malnutrition affects up to 75% of children with cancer, primarily due to low dietary intake and excessive disease-related energy expenditure [5]. Some studies have explored nutritional interventions for pediatric patients with cancer, focusing on hospital-based nutritional support, targeted dietary modifications, and structured exercise regimens [6]. However, these interventions have often yielded unsatisfactory outcomes. The ineffectiveness of many nutritional interventions can be attributed to diminished appetite and inadequate food intake in children [7]. Appetite, as the primary regulator of food intake, is crucial for improving nutrition [8]. Therefore, developing strategies to stimulate appetite and promote food intake is essential to prevent malnutrition and maintain quality of life in pediatric patients with cancer.

Pediatric patients with cancer often experience appetite loss [7], and social media content such as mukbang is increasingly being explored by children to boost their food intake. Mukbang, a globally recognized phenomenon, is an online culinary spectacle that captivates viewers with a fusion of auditory and visual experiences and fosters dynamic, interactive dialogue between the host and the audience [9]. Food-related visual and auditory stimuli have been found to enhance the exploration of various sensory dimensions, including those linked to physical tasting encounters [10,11]. Evidence shows that individuals experience positive emotional changes and report diverse taste sensations when exposed to various mukbang videos [12]. A recent scoping review also emphasized that mukbang may have positive effects on viewers’ appetites, food choices, and psychological well-being [11]. Mukbang watching is a deliberate, self-motivated choice among children to invigorate their appetites, reflecting a growing acceptance of interactive culinary experiences.

However, evidence has also linked problematic mukbang watching to disordered eating behaviors, showing a significant positive correlation [13]. A previous qualitative study further explored the psychological and physiological experiences of youth watching mukbang, finding that while it may alleviate mental health concerns and stimulate appetite, long-term watching might have adverse health effects [14]. Given the uncertainty surrounding the impact of mukbang-watching behavior among pediatric patients with cancer, further research is needed.

Appetite’s transient and subjective nature suggests that cross-sectional methods may be subject to recall biases. An intensive longitudinal approach, tracking appetite and watching habits among pediatric patients with cancer in natural settings, provides more insights than retrospective studies. Therefore, this study aimed to establish evidence for the potential of mukbang as an intervention to enhance nutrition and appetite in children with cancer by exploring its impact on the appetite, nutrition, and quality of life of pediatric patients through an intensive longitudinal study.

## Methods

### Study Design

We conducted an intensive longitudinal study using ecological momentary assessment [15] to assess the association between mukbang watching and appetite, nutritional status, and quality of life in children with cancer undergoing radiotherapy or chemotherapy. Intensive longitudinal designs rely on high-frequency repeated assessments over short periods to capture within-person fluctuations. Nutritional status and quality of life were measured at baseline and at the end of treatment. Mukbang watching and appetite were assessed 15 times over 5 consecutive days, with assessments conducted 3 times daily at mealtimes to capture real-time changes. This design minimized recall bias [16] and accurately reflected the transient nature of appetite. The protocol has been prospectively registered on ClinicalTrials.gov (NCT05493020).

### Participants

Recruitment was conducted between September 2022 and June 2023 using convenience sampling. Children hospitalized in the pediatric oncology wards of 2 tertiary hospitals in China were invited to participate. Eligible participants met the following criteria: (1) aged 3 to 18 years; (2) diagnosed with cancer according to the *Notice on Diagnosis and Treatment Specifications for 12 Pediatric Hematological and Malignancy Diseases (2021 Edition)*, issued by the General Office of the China National Health and Health Commission [17]; and (3) had received at least 1 session of radiotherapy or chemotherapy.

Exclusion criteria included (1) severe cardiopulmonary disorders, serious infections, significant organ damage, genetic metabolic disorders, immune deficiencies, or mental illnesses; (2) inability to watch or listen to videos due to physical dysfunction; and (3) restricted diet due to illness or treatment factors. Dropout criteria included transfer or death, physical weakness preventing participation, and discontinuation of radiation or chemotherapy for any reason during the study.

### Sample Size

The sample size was calculated using an online calculator developed for intensive longitudinal studies based on the framework described by Pirla et al [18]. Using the affect dynamics measure with an effect size of 0.33, a power of 0.9, and an α level of .05 [19], a minimum of 110 participants was required for 15 repeated measures. Accounting for a 10% dropout rate at the end of follow-up, this study required at least 122 participants.

### Measures

#### Overview

A structured questionnaire was used to collect data. Demographic details (age and sex), disease characteristics (diagnosis, treatment type, and phase), and nutritional indicators (serum albumin, hemoglobin, and arm circumference) were obtained from the medical records of the patients. Mukbang watching behaviors, therapy side effects, appetite, nutritional status, and quality of life were evaluated using various questionnaires and scales. The selected variables assess the pathway from behavioral triggers (mukbang watching) to health outcomes (appetite, nutrition, and quality of life) according to a previous qualitative study [14]. All these instruments were utilized with formal permission from their respective developers or copyright holders.

#### Characteristics of Mukbang-Watching Behavior

Mukbang-watching behaviors were recorded using a self-designed questionnaire adapted from our prior qualitative research on Chinese youth [14]. It included details on watching duration, frequency, preferences, and experiences during mukbang sessions. To ensure the clarity and feasibility of the items, the tool was pilot-tested with 5 pediatric patients, and the questionnaire was further refined based on their feedback before formal administration.

#### Therapy-Related Symptom Checklist for Children

Therapy-related symptoms were assessed using the 26-item TRSC-C (Therapy-Related Symptom Checklist for Children) (Checklist 1) [20]. Each item is rated on a 5-point scale, with a higher score indicating more severe symptoms. The scale has shown good internal consistency (Cronbach *α*=0.87) among Chinese children [21].

#### Cancer Appetite and Symptom Questionnaire

Appetite was measured using the 12-item Cancer Appetite and Symptom Questionnaire (CASQ). The total score of the questionnaire is 48 points [22], with lower total scores indicating poor appetite. The Chinese version of the CASQ demonstrated good split-half reliability, with a score of 0.85 [23].

#### Subjective Global Nutritional Assessment

Subjective global nutritional assessment was adopted to measure the nutritional status of participants [24]. The standard includes (1) well-nourished, when the child is growing normally, with adequate food consumption and without gastrointestinal symptoms; (2) moderately malnourished, when the patient shows signs of weight loss or loss of food consumption, functional capacity, and reduced muscle mass, demonstrating nutritional status impairment when it was previously normal; and (3) severely malnourished, when the child has progressive malnutrition, with weight loss, reduction of muscle and fat mass, and loss of food consumption. Participants were classified as well-nourished, moderately malnourished, or severely malnourished. The Chinese version of the subjective global nutritional assessment demonstrated a substantial Cronbach α coefficient of 0.736 [25].

#### Pediatric Quality of Life Inventory Measurement Models 3.0

Quality of life was measured using the 27-item Pediatric Quality of Life Inventory (PedsQL) 3.0 Cancer Module [26]. Each item is rated on a 5-point Likert scale. Items were reverse-scored and linearly transformed to a 0‐100 scale (0=100, 1=75, 2=50, 3=25, and 4=0) following the standard PedsQL scoring protocol. The total score was calculated as the mean of all items, with higher scores representing a better quality of life. The PedsQL has demonstrated reliability in evaluating the quality of life in childhood cancer survivors, exhibiting Cronbach α coefficients between 0.87 and 0.95 across its subscales [27].

### Data Collection

Baseline data were collected on demographics, disease characteristics, therapy-related symptoms, mukbang-watching behavior, appetite, nutritional status, and quality of life. Participants and their guardians were given a self-recorded brochure (Multimedia Appendix 1) to track their appetite and mukbang-watching behavior during meals for 5 consecutive days starting from the first day of radiotherapy or chemotherapy. To maximize data completeness, research staff performed a brief quality check upon collection to ensure no mealtimes were inadvertently skipped. If any missing entries were identified, parents were invited to provide the information while their memory was still fresh. Nutritional status and quality of life were assessed at the end of the current treatment phase. The schedule for data collection and the flowchart of sample selection are presented in Multimedia Appendix 2.

### Statistical Analysis

Categorical variables are shown as frequencies and percentages, while continuous variables and normally distributed data are presented as mean (SD). The Wilcoxon and chi-square tests were used to identify factors differentiating the mukbang watching and nonwatching classes.

The frequency of mukbang-watching behavior was classified using latent class analysis (LCA) with R software (version 4.2.0; R Foundation for Statistical Computing). Models with 1 to 5 latent classes were repeatedly fitted, increasing the number of latent classes in a stepwise manner. The models were evaluated using log likelihood, Akaike information criterion, adjusted Bayesian information criterion, entropy, and the Vuong-Lo-Mendell-Rubin ratio test. Lower log likelihood, Bayesian information criterion, and Akaike information criterion values indicate a better model fit [28], and relative entropy values greater than 0.80 indicate sufficient certainty in classification [28]. The features of the latent classes were compared using a chi-square test. Subsequently, unadjusted and adjusted generalized estimating equation (GEE) models were used to explore the association between mukbang-watching behavior and changes in appetite, nutritional status, and quality of life over time. This approach was chosen to effectively account for the correlation between repeated measures and to provide robust population-averaged estimates for the longitudinal data.

Additionally, to assess the robustness of our primary findings against baseline appetite imbalances, we conducted a sensitivity analysis by stratifying the participants based on CASQ scores into 3 groups: low (0‐16), medium (17-32), and high (33-48) baseline appetite. Given the restricted distribution of actual scores, in our sample, no participants fell into the low appetite category at baseline. We conducted the sensitivity analysis within the medium and high baseline appetite subgroups and re-evaluated the associations between mukbang watching and appetite, nutrition, and quality of life using adjusted GEE models.

Except for LCA, all statistical analyses were performed using SPSS (version 26.0; IBM Corp). Statistical significance was set at 2-sided *P*<.05. No missing values were found in this study due to careful checking of the data for each collection.

### Ethical Considerations

Ethical approval for the study was obtained from the institutional review board of the School of Nursing, Sun Yat-sen University, following the Declaration of Helsinki (reference: L2022SYSU-HL-089). Written informed consent was obtained from all participants and their guardians prior to their participation, after they were fully informed of the study’s purpose and procedures. To ensure privacy and confidentiality, all personal identifiers were removed, and a unique study identification number was assigned to each participant for data entry, management, and analysis.

## Results

The study enrolled 179 eligible participants, with no dropouts during the follow-up period. As shown in Table 1, participants had a mean age of 7.7 (SD 3.7) years, with 60.3% (n=108) being male and 65.4% (n=117) diagnosed with leukemia. Most (n=67, 37.4%) were in their first phase of treatment. At baseline, participants reported an average of 4.1 (SD 4.0) days of appetite loss during their last treatment, and 83.8% (n=150) demonstrated adequate nutritional status. The mean quality of life score was 72.8 (SD 12.5).

**Table 1.** Demographic and disease-related data of participants at baseline (N=179).

Item	Values			*P* value
	Total (N=179)	Watch mukbang (n=114)	Do not watch mukbang (n=65)	
				.42
Age (y)^a^, mean (SD)	7.7 (3.7)	7.5 (3.7)	8.0 (3.7)	
3, n (%)	82 (45.8)	55 (48.2)	27 (41.5)	
7, n (%)	32 (17.9)	17 (14.9)	15 (23.1)	
10‐18, n (%)	65 (36.3)	42 (36.8)	23 (35.4)	
Sex, n (%)				.31
Male	108 (60.3)	72 (63.2)	36 (55.4)	
Female	71 (39.7)	42 (36.8)	29 (44.6)	
Height (cm), mean (SD)	125.6 (25.1)	125.4 (26.2)	125.8 (23.5)	.91
Arm circumference (cm), mean (SD)	17.5 (3.8)	17.5 (3.9)	17.5 (3.7)	.93
Weight (kg), mean (SD)	27.3 (14.7)	27.7 (15.9)	26.5 (12.3)	.58
Disease diagnosis, n (%)				.009
Leukemia	117 (65.4)	83 (72.8)	34 (52.3)	
Lymphoma	16 (8.9)	8 (7)	8 (12.3)	
Sarcoma	16 (8.9)	8 (7)	8 (12.3)	
Neuroblastoma	10 (5.6)	8 (7)	2 (3.1)	
* *Others^b^	20 (11.2)	7 (6.1)	13 (20)	
Treatment phase, n (%)				>.99
I	67 (37.4)	43 (37.7)	24 (36.9)	
II	57 (31.8)	36 (31.6)	21 (32.3)	
III	31 (17.3)	20 (17.5)	11 (16.9)	
IV	24 (13.4)	15 (13.2)	9 (13.8)	
Albumin (g/L)^c^, n (%)				.04
<40	82 (45.8)	59 (51.8)	23 (35.4)	
40‐55	97 (54.2)	55 (48.2)	42 (64.6)	
Hemoglobin (g/L)^d^, n (%)				.02
<110	152 (84.9)	102 (89.5)	50 (76.9)	
110‐160	27 (15.1)	12 (10.5)	15 (23.1)	
Duration of the loss of appetite during the last therapy day, mean (SD)	4.1 (4.0)	3.9 (4.0)	4.5 (4.1)	.35
Watch other kinds of videos before or during meals (yes), n (%)	127 (70.9)	90 (70.9)	37 (29.1)	.002
Playing video games before or during meals (yes), n (%)	73 (40.8)	47 (64.4)	26 (35.6)	.87
TRSC-C^e^, mean (SD)	9.4 (6.7)	8.9 (5.4)	10.2 (8.5)	.22
Appetite^f^, mean (SD)	30.4 (3.8)	31.4 (3.6)	28.7 (3.6)	<.001
Nutritional status (adequate nutrition)^g^, n (%)	150 (83.8)	99 (86.8)	51 (78.5)	.14
Quality of life^h^, mean (SD)	72.8 (12.5)	74.4 (11.3)	70.1 (14.0)	.08
The frequency of watching mukbang^i^, n (%)				
Less than once a week	—^j^	12 (10.5)	—	—
Once a week-once a day	—	39 (34.2)	—	—
Once a day-once a meal	—	48 (42.1)	—	—
More than one meal	—	15 (13.2)	—	—
The duration of watching mukbang^i^, n (%)				
Less than half an hour	—	58 (50.9)	—	—
Half an hour-an hour	—	38 (33.3)	—	—
More than an hour	—	18 (15.8)	—	—
The time point for watching mukbang^k^*,* n (%)				
Eating	—	51 (44.7)	—	—
Nauseated	—	5 (4.4)	—	—
Bored	—	90 (78.9)	—	—
Insomnia	—	2 (1.8)	—	—
The experience of watching mukbang^i^^k^, n (%)				
Increased appetite	—	51 (44.7)	—	—
Decreased nausea	—	4 (3.5)	—	—
Increased pleasure	—	85 (74.6)	—	—
Increased contentment	—	30 (26.3)	—	—

a Categorized according to the Erikson stages of development: 3-6 years (preschool), 7-9 years (middle school), and 10-18 years (adolescence).

b Medulloblastoma, hepatoblastoma, nephroblastoma, teratoma, intracranial nongerm cell tumors, left ovarian yolk sac tumor, Langerhans cell histiocytosis, myelodysplastic syndrome, malignant mesenchymal tumors, hemophagocytic syndrome, and sellar region low-grade glioma.

c Pediatric reference interval for albumin: <40 g/L (low), 40‐55 g/L (normal), and >55 g/L (high).

d Pediatric reference interval for hemoglobin: <110 g/L (low), 110‐160 g/L (normal), and >160 g/L (high).

e TRSC-C: Therapy-Related Symptom Checklist for Children. This checklist aimed to enhance comprehension of the specific needs of children and comprises 26 items to cover therapy-related symptoms. Each item is assessed using a numerical scale ranging from 0 to 4, where higher scores correspond to a more pronounced manifestation of symptoms.

f Appetite of children with cancer was measured by the Cancer Appetite and Symptom Questionnaire. A lower total score indicates a worse appetite in a patient.

g Nutritional status was measured by using the subjective global nutritional assessment.

h Quality of life was measured by using the Pediatric Quality of Life Inventory (PedsQL) 3.0 Cancer Module. Higher scores indicate a better quality of life.

i Only children who watched mukbang were included (n=114).

j Not applicable.

k More than 1 answer is possible.

Regarding mukbang watching habits, 63.7% (114/179) of participants reported watching mukbang. Among these viewers, 42.1% (48/114) watched once daily per meal, and 50.9% (58/114) watched for less than half an hour. Notably, a high proportion engaged in mukbang watching due to boredom (90/114, 78.9%) and reported an enhanced sense of pleasure while watching (74.6%, 85/114). Further details regarding the demographic and disease-related characteristics of the participants are presented in Table 1.

The results of the LCA demonstrated that a 4-class model best fits the data, as indicated by the fit statistics shown in Table 2. This model provided a better fit than the 5-class model, with an entropy of 0.935, indicating good separation between the classes. The average posterior probabilities for the classes were 0.84 (class 1), 0.88 (class 2), 0.86 (class 3), and 0.87 (class 4), suggesting minimal classification uncertainty. Classes 1 (C1) to 4 (C4) comprised 66 (36.9%), 26 (14.5%), 51 (28.5%), and 36 (20.1%) of the 179 participants, respectively.

**Table 2.** Model fit statistics for latent class analysis regarding watching mukbang behavior (N=179).

Classes	LL^a^	AIC^b^	BIC^c^	Entropy	VLMR^d^
1	−1934.317	3928.635	4024.256	—^e^	—
2	−1327.031	2776.061	2970.492	0.903	<.001
3	−1256.492	2696.985	2990.224	0.931	<.001
4	−1214.411	2674.822	3066.871	0.935	<.001
5	−1188.508	2685.016	3175.873	0.924	<.001

a LL: log likelihood.

b AIC: Akaike information criterion.

c BIC: Bayesian information criterion.

d VLMR: Vuong-Lo-Mendell-Rubin likelihood ratio test.

e Not applicable.

**Figure 1.**
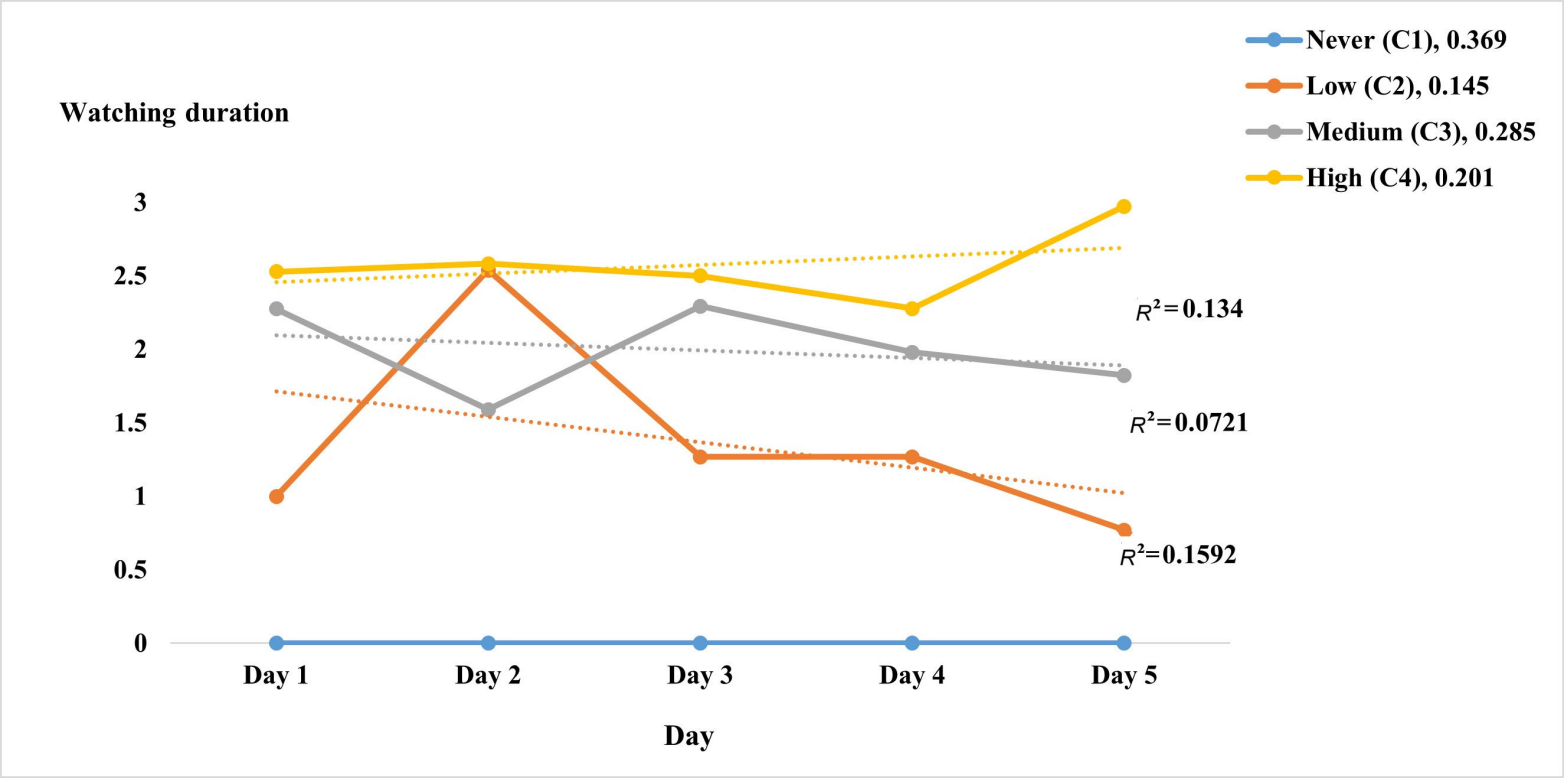
Latent class analysis for the frequency of watching mukbang (N=179). The y-axis displays the daily average of participants’ mukbang watching duration per meal, derived from the original categorical codes (0 for no watching, 1 for less than 0.5 h, 2 for 0.5 to 1 h, and 3 for more than 1 h).

Figure 1 illustrates mukbang watching frequency, sorted by the degree of separation between classes. C1 was defined by the absence of mukbang watching among participants at baseline and throughout the follow-up period. For simplicity, the 4 classes were referred to as never (C1), low (C2), medium (C3), and high (C4) frequencies of mukbang watching.

Baseline demographic and disease characteristics of participants across the 4 classes are detailed in Table 3.

**Table 3.** Demographic and disease-related data of 4-class participants at baseline (N=179).

Item	Values				
	Never (n=66)	Low (n=26)	Medium (n=51)	High (n=36)	*P* value
					.36
Age (y)^a^, mean (SD)	7.8 (3.7)	8.1 (3.9)	8.1 (3.7)	6.8 (3.5)	
3, n (%)	30 (45.5)	10 (38.5)	22 (43.1)	20 (55.6)	
7, n (%)	14 (21.2)	5 (19.2)	7 (13.7)	6 (16.7)	
10‐18, n (%)	22(33.3)	11(42.3)	22(43.1)	10(27.8)	
Sex, n (%)					.61
Male	37 (56.1)	16 (61.5)	30 (58.8)	25 (69.4)	
Female	29 (43.9)	10 (38.5)	21 (41.2)	11 (30.6)	
Height, cm, mean (SD)	124.9 (23.5)	129.4 (29.6)	129.3 (26.8)	118.7 (21.5)	.22
Arm circumference (cm), mean (SD)	17.5 (3.6)	18.3 (4.6)	18.0 (4.1)	16.2 (2.9)	.10
Weight (kg), mean (SD)	26.1 (12.4)	31.1 (17.7)	29.9 (17.9)	23.1 (9.7)	.08
Disease diagnosis, n (%)					.02
Leukemia	37 (56.1)	20 (76.9)	36 (70.6)	24 (66.7)	
Lymphoma	8 (12.1)	2 (7.7)	4 (7.8)	2 (5.6)	
Sarcoma	7 (10.6)	0 (0)	6 (11.8)	3 (8.3)	
Neuroblastoma	1 (1.5)	1 (3.8)	2 (3.9)	6 (16.7)	
Others^b^	13 (19.7)	3 (11.5)	3 (5.9)	1 (2.8)	
Treatment phase, n (%)					.08
I	23 (34.8)	14 (53.8)	14 (27.5)	16 (44.4)	
II	22 (33.3)	5 (19.2)	16 (28.1)	14 (38.9)	
III	13 (19.7)	5 (19.2)	13 (25.5)	0 (0)	
IV	8 (12.1)	2 (7.7)	8 (15.7)	6 (16.7)	
Albumin (g/L)^c^, n (%)					.43
<40	25 (37.9)	13 (50)	25 (49)	19 (23.2)	
40‐55	41 (62.1)	13 (50)	26 (51)	17 (47.2)	
Hemoglobin (g/L)^d^, n (%)					.17
<110	51 (77.3)	22 (84.6)	46 (90.2)	33 (91.7)	
110‐160	15 (22.7)	4 (15.4)	5 (9.8)	3 (8.3)	
Duration of the loss of appetite during the last therapy (day), mean (SD)	4.7 (4.7)	3.6 (4.6)	3.3 (3.1)	4.3 (3.2)	.24
Watching other kinds of videos before or during meals (yes), n (%)	36 (54.5)	18 (69.2)	45 (88.2)	28 (77.8)	.001
Playing video games before or during meals (yes), n (%)	26 (39.4)	14 (53.8)	22 (43.1)	11 (30.6)	.31
TRSC-C^e^, mean (SD)	10.8 (8.7)	7.9 (5.4)	8.8 (5.8)	8.8 (3.8)	.18
Appetite^f^, mean (SD*)*	28.6 (3.6)	31.4 (3.2)	31.9 (3.7)	30.9 (3.5)	<.001
Nutritional status (adequate nutrition)^g^, n (%)	51 (77.3)	22 (84.6)	45 (88.2)	32 (88.9)	.22
Quality of life^h^, mean (SD)	69.7 (14.1)	74.2 (10.0)	77.1 (11.6)	71.5 (10.8)	.02

a Categorized according to the Erikson stages of development: 3‐6 years (preschool), 7‐9 years (middle school), and 10‐18 years (adolescence).

b Medulloblastoma, hepatoblastoma, nephroblastoma, teratoma, intracranial nongerm cell tumors, left ovarian yolk sac tumor, Langerhans cell histiocytosis, myelodysplastic syndrome, malignant mesenchymal tumors, hemophagocytic syndrome, and sellar region low-grade glioma.

c Pediatric reference interval for albumin: <40 g/L (low), 40‐55 g/L (normal), and >55 g/L (high).

d Pediatric reference interval for hemoglobin: <110 g/L (low), 110‐160 g/L (normal), and >160 g/L (high).

e TRSC-C: Therapy-Related Symptom Checklist for Children. Higher scores correspond to a more pronounced manifestation of symptoms.

f Appetite of children with cancer was measured by the Cancer Appetite and Symptom Questionnaire. A lower total score indicates a worse patient’s appetite.

g Nutritional status was measured by the subjective global nutritional assessment.

h Quality of life was measured by the Pediatric Quality of Life Inventory (PedsQL) 3.0 Cancer Module. Higher scores indicate better quality of life.

At baseline, appetite and quality of life significantly differed among the 4 classes, while no significant baseline differences were observed in nutritional status. Longitudinal analysis (Table S1 in Multimedia Appendix 3) indicated that while participants across all 4 classes numerically improved in quality of life compared to baseline, the extent of this improvement varied across classes, highlighting heterogeneity in the observed changes. Meanwhile, overall nutritional status among the 4 classes exhibited a decreasing trend.

As shown in Table 4, compared to the baseline, there were significant differences among the 4 classes in appetite and nutritional status at the end of treatment (all *P*s<.001). The adjusted appetite GEE model indicated significant effects of time × class in both the medium class (β=0.08, 95% CI 0.02‐0.13; *P*=.006) and the high class (β=0.10, 95% CI 0.04‐0.17; *P*=.003). Both the medium class (β=19.18, 95% CI 7.84‐30.53; *P*=.001) and the high class (β=13.63, 95% CI 1.15‐26.12; *P*=.03) showed a significantly higher quality of life compared to the never classes. However, a significant decline in quality of life was also observed in the low class (β=−7.58, 95% CI −14.78 to −0.37; *P*=.04) and the medium class (β=−14.00, 95% CI −22.06 to −5.94; *P*=.001). Changes in appetite, quality of life, and adequate nutrition at baseline (T_0_) and during follow-up (T_1_-T_end_) in the 4 classes, based on unadjusted GEE models, are shown in Table S2 in Multimedia Appendix 3.

**Table 4.** Changes in appetite, quality of life, and adequate nutrition at baseline (T_0_) and during follow-up (T_1_-T_end_) in 4 classes based on adjusted generalized estimating equation models.

Item	Values^a^	*P* value
Appetite^b^, adjusted β (95% CI)		
Time	−0.28 (−0.30 to −0.25)	<.001
Class (reference: never)		
Low	0.07 (−0.50 to 0.65)	.81
Medium	0.33 (−0.13 to 0.80)	.16
High	0.01 (−0.54 to 0.55)	.98
Time × class (reference: time × never)		
Time × low	0.03 (−0.04 to 0.10)	.45
Time × medium	0.08 (0.02 to 0.13)	.006
Time × high	0.10 (0.04 to 0.17)	.003
Nutrition status^b^*,* adjusted OR (95% CI)		
Time	0.20 (0.11 to 0.37)	<.001
Class (reference: never)		
Low	0.45 (0.04 to 4.78)	.51
Medium	0.98 (0.13 to 7.67)	.99
High	0.94 (0.11 to 7.93)	.95
Time × class (reference: time × never)		
Time × low	1.71 (0.54 to 5.46)	.36
Time × medium	1.48 (0.50 to 4.38)	.48
Time × high	1.91 (0.63 to 5.79)	.25
Quality of life^c^, adjusted β (95% CI)		
Time	4.70 (−0.34 to 9.74)	.07
Class (reference: never)		
Low	9.81 (1.12 to 20.75)	.08
Medium	19.18 (7.84 to 30.53)	.001
High	13.63 (1.15 to 26.12)	.03
Time × class (reference: time × never)		
Time × low	−7.58 (−14.78 to −0.37)	.04
Time × medium	−14.00 (−22.06 to −5.94)	.001
Time × high	−8.01 (−16.34 to 0.32)	.06

a β coefficients were reported for appetite and quality of life; odds ratios were reported for nutritional status.

b Adjusted for age, gender, disease diagnosis, treatment phase, playing video games before or during meals, watching other kinds of videos before or during meals, baseline appetite, and TRSC-C (Therapy-Related Symptom Checklist for Children) score.

c Adjusted for age, gender, disease diagnosis, treatment phase, playing video games before or during meals, watching other kinds of videos before or during meals, baseline appetite, TRSC-C score, and quality of life.

As shown in Table S3 in Multimedia Appendix 3, the sensitivity analysis stratified by baseline appetite yielded results consistent with those of the main analysis. Regarding appetite, the adjusted GEE model indicated significant interaction effects in both the medium (β=.07, 95% CI 0.01‐0.13; *P*=.03) and high baseline appetite subgroups (β=.16, 95% CI 0.05‐0.28; *P*=.005). For quality of life, the medium baseline appetite subgroup showed significant class differences (medium: β=19.18, 95% CI 7.84‐30.53, *P*=.001; high: β=13.63, 95% CI 1.15‐26.12, *P*=.03) and significant interaction effects (low: β=−7.58, 95% CI −14.78 to −0.37, *P*=.04; medium: β=−14.00, 95% CI −22.06 to −5.94, *P*=.001). In contrast, the high baseline appetite subgroup only exhibited a significant decline over time (β=−8.72, 95% CI −16.73 to −0.71; *P*=.03).

## Discussion

This study explored the associations between mukbang watching and appetite, nutrition, and quality of life in pediatric patients with cancer using an intensive longitudinal design. These results may provide preliminary evidence for appetite-related symptom management in pediatric patients with cancer.

Reflecting on a broader trend, our study found that most pediatric patients (63.7%) engaged in mukbang, aligning with recent national data showing that 61.8% and 65.1% of junior and senior high school students in China actively use short video platforms, and 72.1% and 77% engage with social media, respectively [29]. In our research, a prevalent pattern of mukbang engagement was observed among pediatric patients, with 89.5% tuning in weekly and 49.1% spending over 30 minutes per session. This trend closely echoes the data of Tayla et al [19], which revealed a committed viewer base, with 34.0% engaging daily for a comparable duration. This trend likely arises from the limited entertainment options and prolonged treatment times experienced by hospitalized children, leading them to seek media that meets their social and companionship needs.

Furthermore, our findings highlighted a notable preference for watching mukbang content while eating, with a considerable proportion of pediatric patients (44.7%) engaging in this behavior—a rate significantly higher than the specific content-based engagement reported in a previous general pediatric study [30]. The combination of video watching with mealtime was notably more common among children with cancer compared to their healthy peers [30]. Guardians often permit pediatric patients with cancer to eat while watching videos as a practical strategy to help mitigate treatment-related issues such as nausea and appetite loss. This approach aligns with broader evidence suggesting that mukbang can enhance mood and provide sensory stimulation [11], potentially serving as a digital tool to overcome clinical barriers to eating.

This study found that appetite in pediatric patients with cancer declined throughout treatment, with significant variations at different times, matching the results of an earlier study [31]. In contrast, the findings also showed that frequent mukbang watching helped mitigate this decline over time. Notably, a graded association was observed, in which higher frequencies of mukbang watching were associated with greater appetite maintenance. This indicates that consistent mukbang watching may be a contributing factor in the stabilization of appetite trajectories. Interestingly, despite the video-watching habits of some participants in the Never class, their appetite levels did not show a positive trend compared to those in other classes actively watching mukbang. The auditory and visual stimuli in mukbang, including the chewing and swallowing sounds and the appealing display of delicious dishes, stimulate the brain to evoke a desire to eat [11]. Frequent exposure to food content on social media can alter eating habits driven by visual attraction [32]. Hunger may activate brain areas, such as the prefrontal and orbitofrontal cortices and the amygdala, when watching food images, sparking intense cravings [33]. Evidence indicates that auditory cues in videos can enhance the perceived flavor of food, which in turn may stimulate the viewer’s appetite [34]. Mukbang creates an enticing ambiance that stimulates appetite.

Beyond sensory stimulation, research has linked loss of appetite in children to various lifestyle factors [35], and mukbang could help address this issue in pediatric patients with cancer. Previous evidence shows that emotional factors often drive food cravings, with eating choices and pace sometimes influenced more by emotions than by physical hunger [36]. The engaging and calming content of mukbang eases the strain of treatment for pediatric patients with cancer, improving their mood and stimulating their appetite. This encourages them to return to these videos during periods of emotional distress or reduced appetite. The graded association suggests that frequent engagement with mukbang potentially amplifies the emotional regulation benefits that alleviate treatment-related appetite loss. Evidence indicates that mukbang, as a type of food-focused media, can address food cravings and anxieties in those on restrictive diets, effectively satisfying appetite-related desires [37]. Mukbang fosters a sense of community for pediatric patients, reducing loneliness and enhancing social support. Its interactive nature strengthens viewer connections by simulating shared dining experiences, which can boost appetite and food consumption.

Despite the observed significant interaction effect on appetite trajectories, no significant changes were found in nutritional status over time. A previous study revealed that mukbang watching is associated with increased food intake [12], though this discrepancy may be explained by the clinical context of cancer treatment. During chemotherapy or other intensive therapies, children often experience treatment-related dietary restrictions, gastrointestinal side effects, mucositis, or infection-control dietary limitations [38], which may prevent increased appetite from translating into greater caloric intake. Moreover, appetite represents a subjective motivational state, whereas nutritional status reflects objective physiological outcomes that may require sustained dietary changes over a longer period to demonstrate measurable improvement [39]. In addition, cancer-related metabolic alterations and systemic inflammation may counteract the potential benefits of increased food desire [40]. Future research could integrate structured nutritional management strategies alongside mukbang watching to examine whether enhanced appetite, when supported by appropriate dietary interventions, contributes to measurable improvements in nutritional outcomes.

A significant baseline difference in quality of life was observed across classes, suggesting that initial well-being and mukbang engagement may be associated. After adjusting for baseline quality of life, our study showed that the observed negative interactions in the low and medium classes indicate a greater decline in quality of life compared to the never class. Sensitivity analyses confirmed the longitudinal trends observed in our study. While the medium baseline appetite subgroup followed a pattern similar to the main analysis, the high baseline appetite subgroup exhibited a distinct decline over time. These results indicate that the association between mukbang watching frequency and quality of life varies according to the patient’s baseline appetite. This discrepancy suggests that the physiological and psychological impact of mukbang watching is sensitive to the patient’s baseline appetite, which may be explained by the psychological frustration arising when enhanced appetite meets the physical constraints of treatment [41]. Specifically, the strong desire for food stimulated by mukbang may conflict with patients’ dietary restrictions or gastrointestinal discomfort. This contrast between virtual enjoyment and physical reality may create a psychological gap and a sense of deprivation, thereby hindering rapid gains in overall well-being [42]. Beyond that, repeated exposure to highly rewarding food cues without actual consumption may increase reward prediction error and emotional dysregulation [43]. Mukbang may also function as a passive coping strategy that temporarily alleviates distress but limits engagement in more adaptive coping behaviors. Future research should further explore the long-term impact of mukbang watching on both physiological and psychological outcomes.

This study had certain limitations, including a small sample size and a short tracking period focusing on a single pediatric oncology treatment cycle. The relatively smaller sample size of the high baseline appetite subgroup (n=59) may limit the robustness of our sensitivity analyses. While the single-center design may limit the generalizability of the findings, it allowed for consistent data collection protocols. Furthermore, although we implemented real-time data verification to minimize measurement bias, the subjective nature of the CASQ scale and the involvement of parents may cause reporting bias, as caregivers’ perceptions may not fully reflect the child’s subjective experience. This study also did not explore the relationship between mukbang content and appetite due to the absence of a standard definition for mukbang. Future qualitative follow-up research is recommended to better understand mukbang watching among pediatric patients with cancer.

Despite its limitations, the findings provide preliminary insights for clinical practice and research implications. Our study represents a novel exploration of the associations between mukbang watching and the appetite, nutritional status, and quality of life of pediatric patients with cancer. By using an intensive longitudinal study, we documented the real-time behaviors and appetite of pediatric patients with cancer watching mukbang, offering valuable insights into their evolving needs throughout treatment. These results provide preliminary empirical evidence suggesting that mukbang could be a potential supportive strategy to manage appetite in this vulnerable population.

Our findings show that frequent mukbang watching is associated with better appetite maintenance in pediatric patients with cancer, particularly in the medium and high classes. However, the longitudinal patterns of quality of life were less favorable, with the low and medium classes exhibiting a slower recovery process compared to the never class. Sensitivity analyses revealed that the association between mukbang watching and quality of life varies by baseline appetite. While the medium baseline appetite subgroup showed significant longitudinal differences, the high baseline appetite subgroup was characterized primarily by a significant decline over time. These results indicate that the association between mukbang watching and quality of life is not uniform but is sensitive to the patient’s initial appetite status. Further research is needed to identify the factors that may contribute to these varying associations and to explore whether mukbang can be optimized as a supportive strategy for pediatric patients with cancer.

## Supplementary material

10.2196/80932 Multimedia Appendix 1 Self-reported brochure.

10.2196/80932 Multimedia Appendix 2 Data collection schedule.

10.2196/80932 Multimedia Appendix 3 Participants’ profiles of appetite, quality of life, and nutritional status at baseline and during follow-up (Table S1), the longitudinal results based on unadjusted generalized estimating equations (Table S2), and adjusted generalized estimating equations stratified by baseline appetite subgroups (Table S3).

10.2196/80932 Checklist 1 TRSC-C checklist.

## Abbreviations

CASQ: Cancer Appetite and Symptom Questionnaire
GEE: generalized estimating equation
LCA: latent class analysis
PedsQL: Pediatric Quality of Life Inventory
SGNA: Subjective Global Nutritional Assessment
TRSC-C: Therapy-Related Symptom Checklist for Children
