# Supplementary material for: Associations Between Mukbang Watching and Appetite, Nutrition, and Quality of Life in Pediatric Patients with Cancer: Intensive Longitudinal Study
Source: J Med Internet Res. 2026 May 22;28:e80932. doi: 10.2196/80932 (PMC13197161; doi:10.2196/80932)

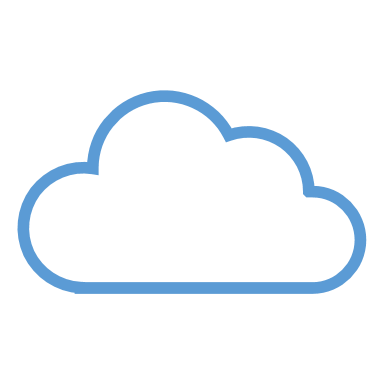


**Food Intake Diary**


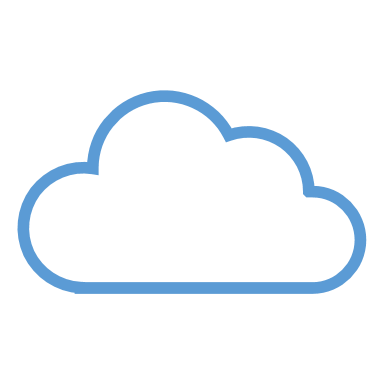


**Dear children and guardians,**

We are currently conducting a study titled "***The Impact of Mukbang Watching Behaviors on the Appetite of Pediatric Oncology Patients During Chemotherapy and Radiotherapy: An Intensive Longitudinal Study.***" We kindly ask for your assistance in observing and recording the child's experience of watching Mukbang videos and their appetite during each of the three daily meals while hospitalized. Your participation in filling out this questionnaire is greatly appreciated. The survey is anonymous, and all data will be used solely for statistical analysis.

Please take a moment to carefully review the items listed below and respond to each question according to the actual circumstances you have experienced.

Thank you in advance for your valuable collaboration and support!

Name:

Number:

Hospital:

Date:

| **DAY__- Breakfast/Lunch/Dinner** |
| --- |
| **Characteristics of watching Mukbang** |
| 1.**Watching Mukbang**: 🞎No 🞎Yes  2. **Duration**:🞎<0.5h 🞎0.5-1h🞎 >1h  3. **Timepoint(multiple-choice)**: 🞎Eating 🞎Nausea 🞎Bored 🞎Sleepless 🞎Others: 4. **Preference**:  5.**Experience(multiple-choice)**:  🞎Enhanced appetite 🞎Diminished nausea 🞎Elevated joy  🞎Augmented fulfillment 🞎Others: |
| **Appetite** |
| **** |


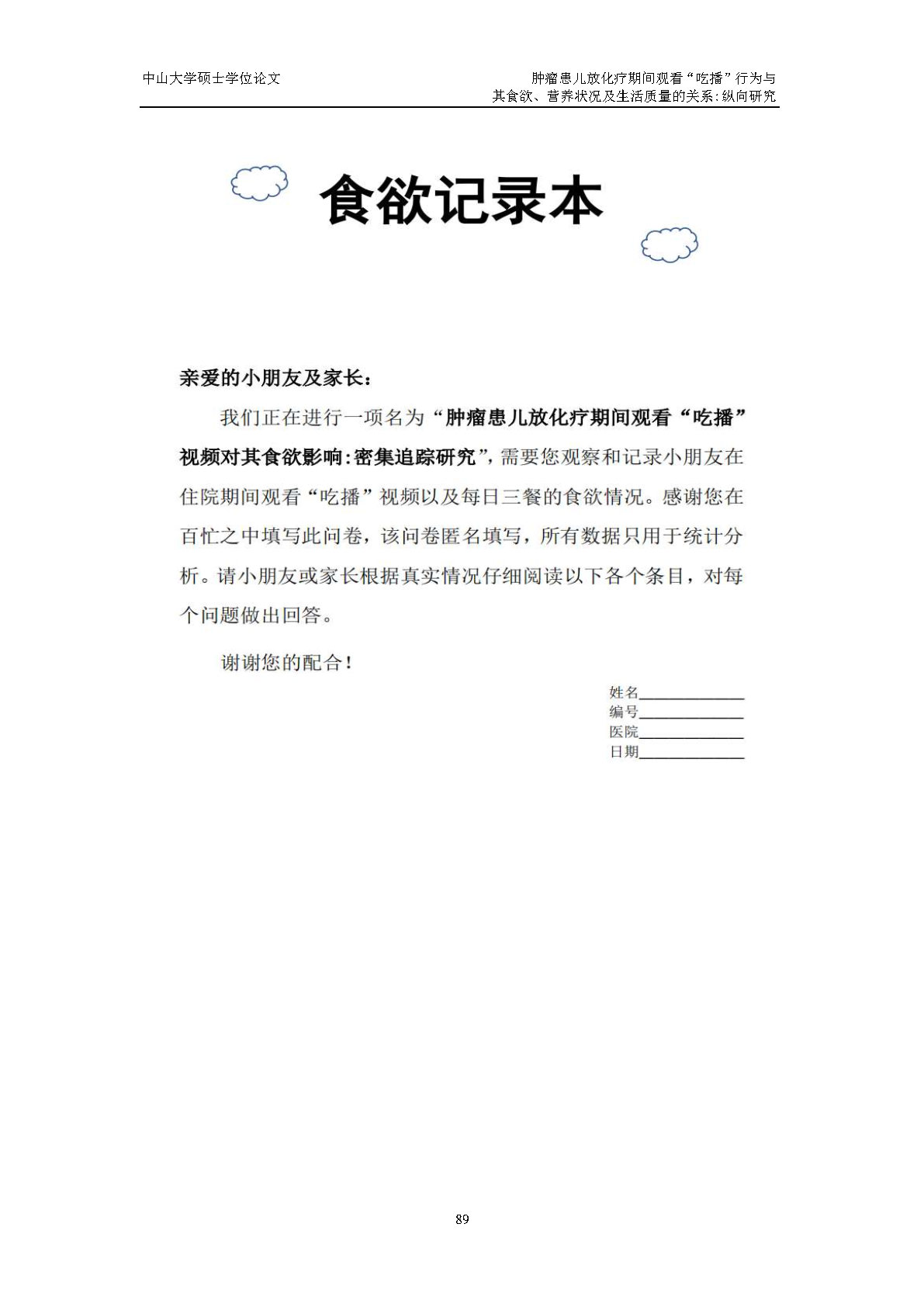


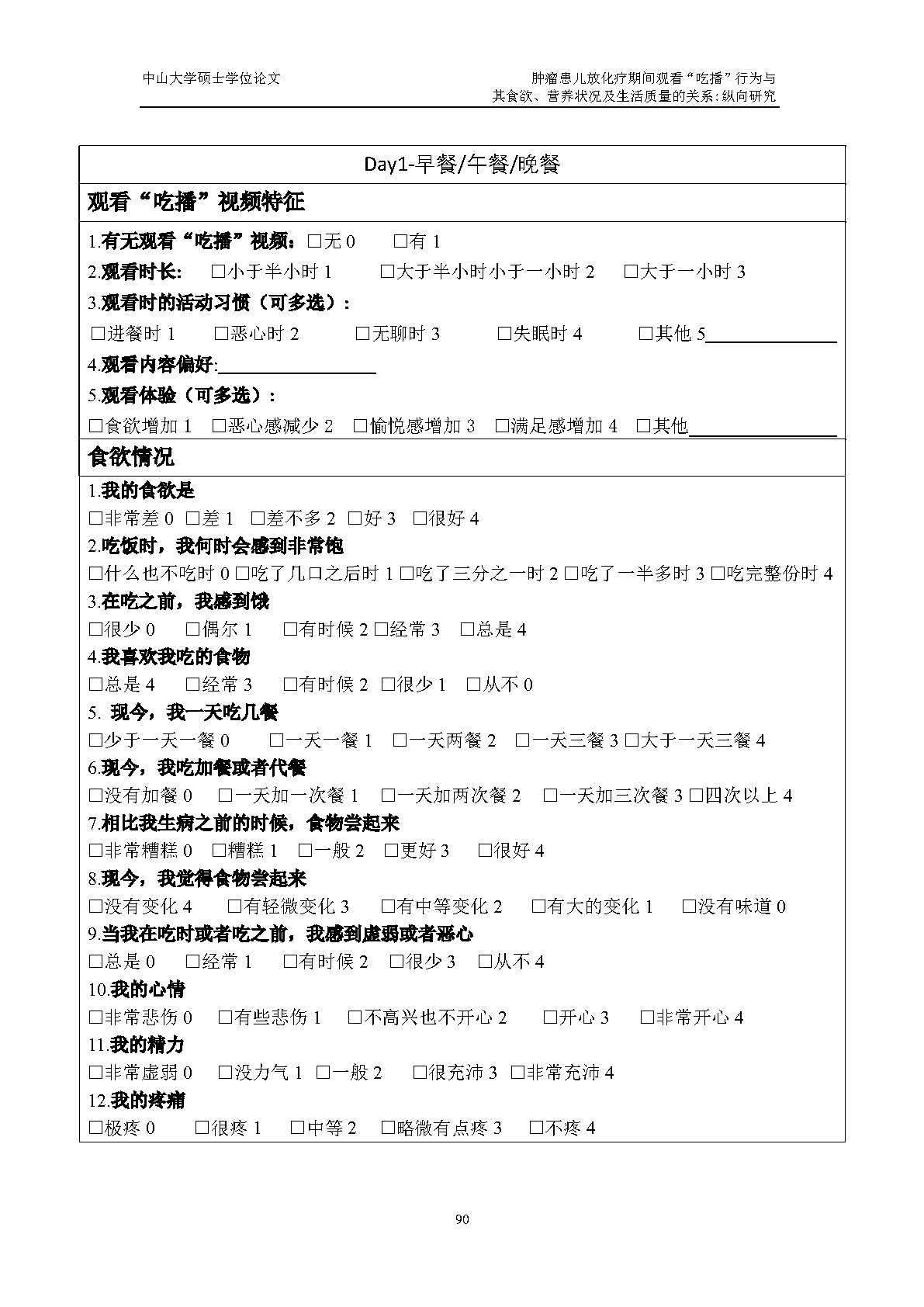

Supplement: Multimedia Appendix 1 [file jmir-v28-e80932-s001.docx]
