# Supplementary material for: Associations Between Mukbang Watching and Appetite, Nutrition, and Quality of Life in Pediatric Patients with Cancer: Intensive Longitudinal Study
Source: J Med Internet Res. 2026 May 22;28:e80932. doi: 10.2196/80932 (PMC13197161; doi:10.2196/80932)
Supplement: Multimedia Appendix 2 [file jmir-v28-e80932-s002.docx]

**Schedule for data collection**

| **Variables** | **Baseline** | **Day1** | **Day2** | **Day3** | **Day4** | **Day5** | **Termination of treatment sessions or**  **hospital discharge** |
| --- | --- | --- | --- | --- | --- | --- | --- |
| **Demographic data** | √ |  |  |  |  |  |  |
| **Disease-related data** | √ |  |  |  |  |  |  |
| **Treatment-related symptoms** | √ |  |  |  |  |  |  |
| **Characteristics of watching Mukbang** | √ | √ | √ | √ | √ | √ |  |
| **Appetite** | √ | √ | √ | √ | √ | √ |  |
| **Nutritional status** | √ |  |  |  |  |  | √ |
| **Quality of life** | √ |  |  |  |  |  | √ |


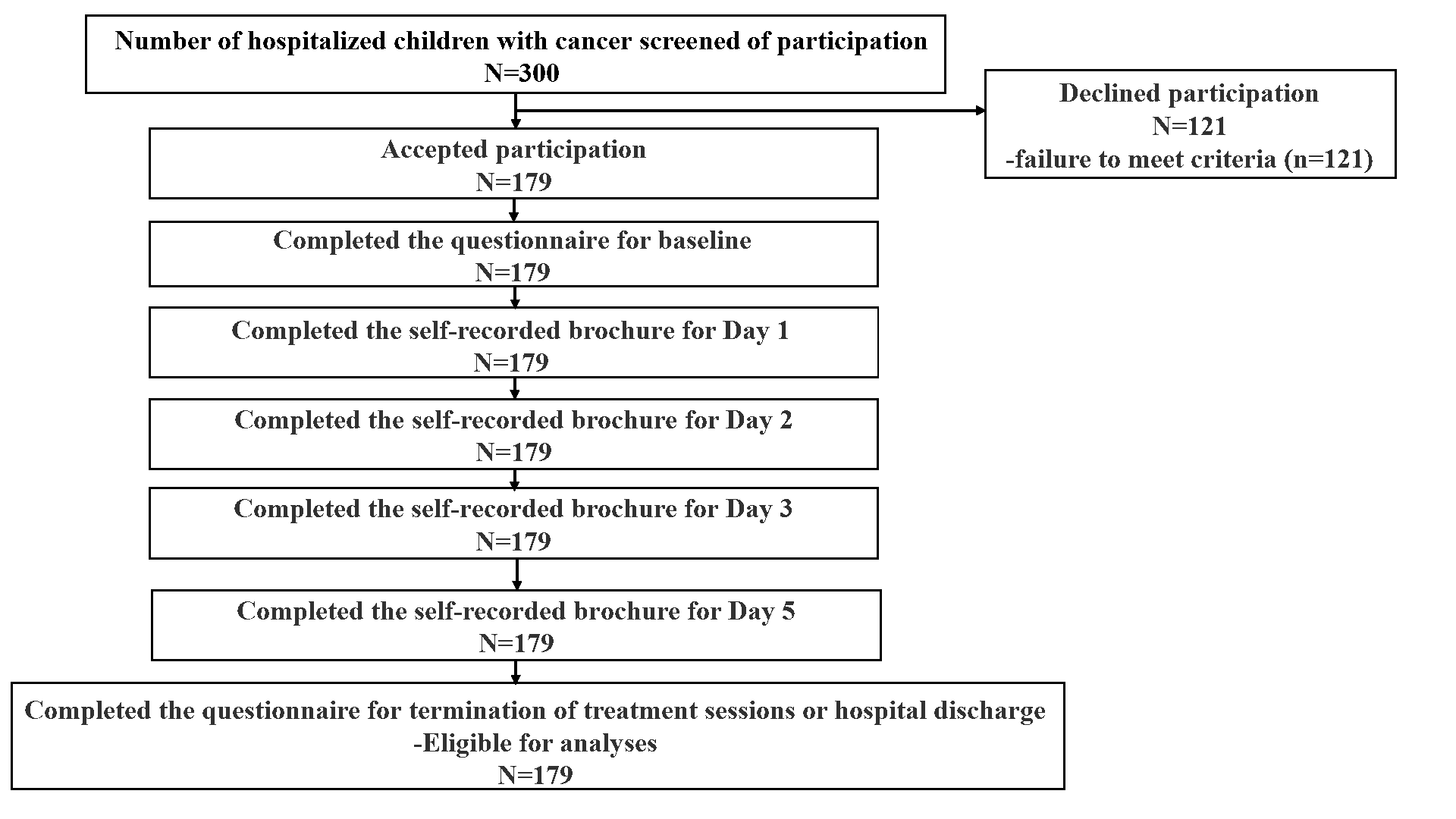


**Flowchart of sample selection**
