# Supplementary material for: Associations Between Mukbang Watching and Appetite, Nutrition, and Quality of Life in Pediatric Patients with Cancer: Intensive Longitudinal Study
Source: J Med Internet Res. 2026 May 22;28:e80932. doi: 10.2196/80932 (PMC13197161; doi:10.2196/80932)
Supplement: Multimedia Appendix 3 [file jmir-v28-e80932-s003.docx]

**Table S1 Participants’ profile of appetite, quality of life, and nutritional status in 4 groups at baseline (T_0_), follow-up (T_1_-T_end_)**

| **Item** | | | **Never**  **(n=66)** | | **Low**  **(n=26)** | **Medium**  **(n=51)** | | | | **High**  **(n=36)** |  |
| --- | --- | --- | --- | --- | --- | --- | --- | --- | --- | --- | --- |
| **Appetite, mean (SD)** | | | | | | | | | | | |
| *T_0_* | 28.6(SD 3.6) | | | 31.4(SD 3.2) | | 31.9(SD 3.7) | | 30.9(SD 3.5) | | |  |
| *T_1_* | 27.8(SD 3.5) | | | 30.6(SD 3.3) | | 31.3(SD 3.8) | | 30.1(SD 3.3) | | |  |
| *T_2_* | 27.8(SD 3.5) | | | 30.0(SD 3.6) | | 31.2(SD 3.8) | | 29.5(SD 3.8) | | |  |
| *T_3_* | 26.7(SD 3.6) | | | 29.8(SD 3.8) | | 31.2(SD 3.9) | | 29.6(SD 3.1) | | |  |
| *T_4_* | 26.5(SD 3.9) | | | 29.6(SD 3.3) | | 30.2(SD 4.1) | | 29.3(SD 3.9) | | |  |
| *T_5_* | 25.8(SD 3.9) | | | 29.9(SD 3.5) | | 29.8(SD 4.3) | | 28.5(SD 4.8) | | |  |
| *T_6_* | 25.5(SD 3.8) | | | 29.2(SD 4.1) | | 29.3(SD 4.1) | | 28.8(SD 4.3) | | |  |
| *T_7_* | 25.4(SD 3.7) | | | 28.1(SD 3.4) | | 29.6(SD 4.4) | | 28.0(SD 4.7) | | |  |
| *T_8_* | 24.7(SD 4.3) | | | 28.2(SD 4.0) | | 29.3(SD 4.7) | | 28.3(SD 4.8) | | |  |
| *T_9_* | 24.3(SD 4.3) | | | 28.2(SD 4.5) | | 29.1(SD 4.8) | | 28.3(SD 5.0) | | |  |
| *T_10_* | 24.7(SD 4.3) | | | 27.4(SD 3.9) | | 28.5(SD 4.3) | | 27.6(SD 4.9) | | |  |
| *T_11_* | 24.3(SD 4.4) | | | 28.1(SD 4.0) | | 29.1(SD 4.7) | | 28.1(SD 5.0) | | |  |
| *T_12_* | 23.9(SD 4.2) | | | 27.9(SD 4.4) | | 28.9(SD 5.0) | | 28.0(SD 5.2) | | |  |
| *T_13_* | 24.4(SD 4.1) | | | 27.5(SD 3.9) | | 28.6(SD 4.3) | | 27.7(SD 4.9) | | |  |
| *T_14_* | 24.0(SD 4.2) | | | 27.3(SD 4.1) | | 28.7(SD 4.8) | | 28.1(SD 5.0) | | |  |
| *T_end_* | 24.3(SD 4.2) | | | 27.4(SD 4.5) | | 29.2(SD 4.9) | | 27.8(SD 4.9) | | |  |
| **Nutrition status (adequate nutrition), N (%)** | | | | | | | | | | |  |
| *T_0_* | | | 51(77.3) | | 22(84.6) | 45(88.2) | | | | 32(88.9) |  |
| *T_end_* | | | 31(47.0) | | 18(69.2) | 37(72.5) | | | | 28(77.8) |  |
| **Quality of life, mean (SD)** | | | | | | | | | | | |
| *T_0_* | | 69.7(SD 14.1) | | | 74.2(SD 10.0) | | 77.1(SD 11.6) | | 71.5(SD 10.8) | |  |
| *T_end_* | | 72.4(SD 12.4) | | | 70.2(SD 15.8) | | 70.2(SD 13.8) | | 70.0(SD 10.2) | |  |

**Table** **S2 Changes in appetite, quality of life, and adequate nutrition at baseline (T_0_) and during follow-up (T_1_-T_end_) in 4 classes based on unadjusted GEE models**

| **Item** | **Crude β/OR (95% CI) *^b^*** | ***P* value** |
| --- | --- | --- |
| **Appetite** |  |  |
| Time | -0.28(-0.32 to -0.25) | <.001 |
| Class *(Reference: Never)* | | |
| *Low* | 3.09(1.62 to 4.57) | <.001 |
| *Medium* | 3.67(2.37 to 4.96) | 0.001 |
| *High* | 2.39(1.02 to 3.76) | <.001 |
| Time×Class *(Reference: Time*×*Never)* | | |
| *Time*×*Low* | 0.03(-0.04 to 0.10) | .44 |
| *Time*×*Medium* | 0.08(0.02 to 0.13) | .006 |
| *Time*×*High* | 0.11(0.04 to 0.17) | .002 |
| **Adequate nutrition *^a^*** |  |  |
| Time | 0.32(0.22 to 0.46) | <.001 |
| Class *(Reference: Never)* | | |
| *Low* | 2.13(0.81 to 5.57) | .12 |
| *Medium* | 2.65(1.25 to 5.60) | .01 |
| *High* | 3.26(1.28 to 8.28) | .01 |
| Time×Class *(Reference: Time*×*Never)* | | |
| *Time*×*Low* | 1.57(0.59 to 4.22) | .37 |
| *Time*×*Medium* | 1.35(0.52 to 3.50) | .53 |
| *Time*×*High* | 1.68(0.65 to 4.33) | .28 |
| **Quality of life *^a^*** |  |  |
| Time | 2.68(-1.92 to 7.28) | .25 |
| Class *(Reference: Never)* | | |
| *Low* | 11.28(0.38 to 22.19) | .04 |
| *Medium* | 16.82(5.90 to 27.74) | .003 |
| *High* | 6.10(-4.62 to 16.82) | .27 |
| Time×Class *(Reference: Time*×*Never)* | | |
| *Time*×*Low* | -6.72(-14.22 to 0.78) | .08 |
| *Time*×*Medium* | -9.41(-16.48 to -2.34) | .01 |
| *Time*×*High* | -4.24(-10.86 to 2.39) | .21 |

Notes:

*^a^* The overall model effect for Time×Class interaction was not significant.

*^b^* β coefficients were reported for appetite and quality of life; ORs were reported for nutritional status.

**Table S3 Changes in appetite, quality of life, and adequate nutrition at baseline (T_0_) and during follow-up (T_1_-T_end_) in 4 classes** **stratified by baseline appetite subgroups based on adjusted GEE models**

|  | **Adjusted β/OR (95% CI) *^d^*** | ***P* value** |
| --- | --- | --- |
| **Medium baseline appetite subgroup (n=120)** | | |
| **Appetite *^a^*** |  |  |
| Time | -0.29(-0.33 to -0.25) | <.001 |
| Class *(Reference: Never)* |  |  |
| *Low* | 0.25(-0.49 to 0.98) | .51 |
| *Medium* | 0.50(-0.04 to 1.04) | .07 |
| *High* | 0.12(-0.56 to 0.80) | .73 |
| Time×Class *(Reference: Time×Never)* | | |
| *Time×Low* | 0.03(-0.04 to 0.10) | .38 |
| *Time×Medium* | 0.07(0.01 to 0.13) | .03 |
| *Time×High* | 0.03(-0.05 to 0.10) | .46 |
| **Nutrition status *^a^*** |  |  |
| Time | 0.16(0.08 to 0.32) | <.001 |
| Class *(Reference: Never)* |  |  |
| *Low* | 1.03(0.05 to 22.41) | .99 |
| *Medium* | 0.73(0.06 to 8.53) | .80 |
| *High* | 0.76(0.05 to 11.31) | .84 |
| Time×Class *(Reference: Time×Never)* | | |
| *Time×Low* | 1.21(0.24 to 6.03) | .82 |
| *Time×Medium* | 1.63(0.42 to 6.32) | .48 |
| *Time×High* | 2.14(0.53 to 8.71) | .29 |
| **Quality of life *^b^*** |  |  |
| Time | 4.70(-0.34 to 9.74) | .07 |
| Class (*Reference: Never*) |  |  |
| *Low* | 9.81(-1.12 to 20.75) | .08 |
| *Medium* | 19.18(7.84 to 30.53) | .001 |
| *High* | 13.63(1.15 to 26.12) | .03 |
| Time×Class *(Reference: Time×Never)* | | |
| *Time×Low* | -7.58(-14.78 to -0.37) | .04 |
| *Time×Medium* | -14.00(-22.06 to -5.94) | .001 |
| *Time×High* | -8.01(-16.34 to 0.32) | .06 |
| **High baseline appetite subgroup (n=59)** | | |
| **Appetite *^a^*** |  |  |
| Time | -0.22(-0.31 to -0.12) | <.001 |
| Class *(Reference: Never)* |  |  |
| *Low* | -0.05(-1.03 to 0.94) | .92 |
| *Medium* | 0.68(-0.12 to 1.49) | .10 |
| *High* | 0.23(-1.03 to 1.49) | .72 |
| Time×Class *(Reference: Time×Never)* | | |
| *Time×Low* | -0.03(-0.18 to 0.12) | .68 |
| *Time×Medium* | 0.04(-0.08 to 0.15) | .55 |
| *Time×High* | 0.16(0.05 to 0.28) | .005 |
| **Nutrition status *^c^*** | - | - |
| **Quality of life *^b^*** |  |  |
| Time | -8.72(-16.73 to -0.71) | .03 |
| Class (*Reference: Never*) |  |  |
| *Low* | -14.27(-31.36 to 2.81) | .10 |
| *Medium* | -14.26(-30.87 to 2.35) | .09 |
| *High* | 2.13(-13.35 to 17.61) | .79 |
| Time×Class *(Reference: Time×Never)* | | |
| *Time×Low* | 8.96(-2.97 to 20.89) | .14 |
| *Time×Medium* | 11.80(-0.67 to 24.27) | .06 |
| *Time×High* | -2.50(-14.07 to 9.06) | .67 |

Notes:

*^a^* Adjusted with age, gender, disease diagnosis, treatment phase, playing video games before/during meals, watch other kinds of videos before/during meals, baseline appetite, and TRSC-C score.

*^b^* Adjusted with age, gender, disease diagnosis, treatment phase, playing video games before/during meals, watch other kinds of videos before/during meals, baseline appetite, TRSC-C score, and quality of life.

*^c^* The GEE model for nutritional status in the high baseline appetite subgroup did not converge due to insufficient sample size and sparse cell counts in certain categories.

*^d^* β coefficients were reported for appetite and quality of life; ORs were reported for nutritional status.
