# Supplementary material for: Associations Between Mukbang Watching and Appetite, Nutrition, and Quality of Life in Pediatric Patients with Cancer: Intensive Longitudinal Study
Source: J Med Internet Res. 2026 May 22;28:e80932. doi: 10.2196/80932 (PMC13197161; doi:10.2196/80932)
Supplement: Checklist 1 [file jmir-v28-e80932-s004.pdf]

Therapy-related symptom Checklist for Children (TRSC-C)

ID # ____________________ Hospital： ______________Date: ____________________

PLEASE **IDENTIFY below** THE PROBLEMS YOU HAVE HAD IMMEDIATELY AFTER AND SINCE YOUR LAST TREATMENT. PLEASE **CIRCLE** HOW SEVERS THE PROBLEM WAS ACOORDING TO THE FOLLOWING SCALE:

0=NO SYMPTOM 1=A LILLTE BIT 2=QUITE A BIT 3= A LOT 4=A WHOLE LOT

EXAMPLE	CIRCLE HOW SEVER				
Pain	0	1	2	③	4
Loss of Appetite [Not feeling hungry ; Eat less; Not feel like eating]	0	1	2	3	4
Nausea [Feel like throwing up; Upset stomach; Stomach hurts]	0	1	2	3	4
Vomiting [Throwing up]	0	1	2	3	4
Weight Loss [Losing weight; feel skinnier]	0	1	2	3	4
Score Mouth [Mouth hurts; Hurts to eat]	0	1	2	3	4
Difficulty Swallowing[Hard to swallow Hurts to swallow]	0	1	2	3	4
Sore Throat [Throat hurts]	0	1	2	3	4
Jaw Pain [Jaw hurts; Hurts to chew or yawn]	0	1	2	3	4
Cough [Coughing much]	0	1	2	3	4
Shortness of Breath [Hard to breath; Breath faster]	0	1	2	3	4
Feeling Sluggish [Feel very tired; Feel lazy]	0	1	2	3	4
Depression [Feel sad a lot; Cry more]	0	1	2	3	4
Difficulty Sleeping [Hard to go to sleep; Hard to stay asleep]	0	1	2	3	4
Fever [Feel very hot; High temperature]	0	1	2	3	4
Bruising [Dark spots on skin; Bruise easily]	0	1	2	3	4
Bleeding [Blood nose; Bleed easily from cuts, scratches, or when brushing teeth]	0	1	2	3	4
Hair Loss [Hair falling out; Losing hair]	0	1	2	3	4
Skin Changes [Skin feels dry or red; Skin feels different]	0	1	2	3	4
Pain [It hurts]	0	1	2	3	4
Numbness in Fingers and Toes [Tingling fingers/toes; Fingers/toes" falling asleep"; Fingers/toes feel cold]	0	1	2	3	4
Constipation [Hard to "pee"]	0	1	2	3	4
Afraid [Feel nervous; worried]	0	1	2	3	4
Headache [Head hurts]	0	1	2	3	4
Irritable [Feel upsets easily; get mad easily]	0	1	2	3	4
Agitation [Feel restless; can not stay still]	0	1	2	3	4
Tripping or Falling	0	1	2	3	4
Other (List)____	0	1	2	3	4
Other (List)____	0	1	2	3	4
Other (List)____	0	1	2	3	4

兒童治療相關症狀檢查表(TRSC-C)

編號#____________醫院_____________日期_____________

在最近一次治療結束后，請<u>立即</u>選出你出現的問題，根據下列量表圈出該問題的嚴重程度

0=沒有 1=有一點 2=較多 3=非常多 4=持續存在

檢查項目	舉例	圈出嚴重程度				
☒	疼痛	0	1	2	③	4
☐	味覺改變[食物味道不同；食物味道奇怪]	0	1	2	3	4
☐	厭食[不覺得飢餓；進食減少；不喜歡進食]	0	1	2	3	4
☐	惡心[想嘔吐；胃部不適；胃痛]	0	1	2	3	4
☐	體重下降[體重減輕；感覺消瘦]	0	1	2	3	4
☐	口瘡[口腔疼痛；進食時口腔疼痛]	0	1	2	3	4
☐	吞咽困難[吞咽困難；吞咽時疼痛]	0	1	2	3	4
☐	咽喉疼痛[感覺咽喉疼痛]	0	1	2	3	4
☐	頜骨疼痛[下巴疼痛；咀嚼或打哈欠時疼痛]	0	1	2	3	4
☐	咳嗽[經常咳嗽]	0	1	2	3	4
☐	呼吸急促[呼吸困難；呼吸加快]	0	1	2	3	4
☐	感覺遲鈍[感覺很疲憊；覺得懶惰]	0	1	2	3	4
☐	抑鬱[經常覺得悲傷；經常哭]	0	1	2	3	4
☐	失眠[入睡困難；易醒]	0	1	2	3	4
☐	發熱[感覺熱；體溫高]	0	1	2	3	4
☐	瘀傷[皮膚出現瘀斑；非常容易碰傷]	0	1	2	3	4
☐	出血[鼻出血；小割傷、劃傷或刷牙時容易出血]	0	1	2	3	4
☐	脫髮[頭髮易於脫落；頭髮減少]	0	1	2	3	4
☐	皮膚改變[皮膚乾燥或變紅；皮膚感覺異常]	0	1	2	3	4
☐	疼痛[感覺疼痛]	0	1	2	3	4
☐	四肢末端感覺麻木[手指/腳趾刺痛；手指/腳趾僵硬；手指/腳趾感覺冰涼]	0	1	2	3	4
☐	便秘[大便排出困難]	0	1	2	3	4
☐	恐懼感[感覺緊張；擔憂]	0	1	2	3	4
☐	口乾[口腔感覺乾燥]	0	1	2	3	4
☐	煩躁[容易煩躁不安；易怒]	0	1	2	3	4
☐	激動[感覺坐立不安；無法安靜下來]	0	1	2	3	4
☐	絆倒或跌倒	0	1	2	3	4
☐	其他[請舉例症狀]________________________	0	1	2	3	4
☐	其他[請舉例症狀]________________________	0	1	2	3	4
☐	其他[請舉例症狀]________________________	0	1	2	3	4
☐	其他[請舉例症狀]________________________	0	1	2	3	4
☐	其他[請舉例症狀]________________________	0	1	2	3	4
